# Supplementary material for: Patients’ experiences of living with low anterior resection syndrome three to six months after colorectal cancer surgery: A phenomenological study
Source: PLoS One. 2024 Jul 10;19(7):e0305212. doi: 10.1371/journal.pone.0305212 (PMC11236105; doi:10.1371/journal.pone.0305212)
Supplement: Attachment — Submitted filename: Response to reviewers.docx [file pone.0305212.s003.docx]

Bergen, Norway

April 28, 2024

PONE-D-23-42953

Patients’ experiences of living with low anterior resection syndrome three to six months after colorectal cancer surgery: A phenomenological study

PLOS ONE

Atalel Fentahun Awedew, MD,MPH, Ass.Prof Surgery
Academic Editor
PLOS ONE

Dear Academic Editor,

We appreciate the valuable and constructive comments on our manuscript “*Patients’ experiences of living with low anterior resection syndrome three to six months after colorectal cancer surgery: A phenomenological study*”. Below, please find item-by-item responses to the Editor and Reviewers’ comments, which are included verbatim. All page and paragraph numbers refer to locations in the revised manuscript. Revisions are in red text in the revised manuscript.

**Journal Requirements**:

When submitting your revision, we need you to address these additional requirements.

1. Please ensure that your manuscript meets PLOS ONE's style requirements, including those for file naming. The PLOS ONE style templates can be found at

<https://journals.plos.org/plosone/s/file?id=wjVg/PLOSOne_formatting_sample_main_body.pdf> and 
<https://journals.plos.org/plosone/s/file?id=ba62/PLOSOne_formatting_sample_title_authors_affiliations.pdf>

**RESPONSE**

We believe we now have followed your requirements.

2. We note that Figure 1 in your submission contain copyrighted images. All PLOS content is published under the Creative Commons Attribution License (CC BY 4.0), which means that the manuscript, images, and Supporting Information files will be freely available online, and any third party is permitted to access, download, copy, distribute, and use these materials in any way, even commercially, with proper attribution. For more information, see our copyright guidelines: <http://journals.plos.org/plosone/s/licenses-and-copyright>.
We require you to either (1) present written permission from the copyright holder to publish these figures specifically under the CC BY 4.0 license, or (2) remove the figures from your submission:

**RESPONSE**

It is correct that in our initial submission we included an image from Keane C, Fearnhead NS, Bordeianou LG, Christensen P, Basany EE, Laurberg S, et al. *International Consensus Definition of Low Anterior Resection Syndrome*. Dis Colon Rectum. 2020;63(3):274-84. We obtained permission from the authors to use this image and included their written consent when submitting our manuscript to PLOS ONE. However, we have come to acknowledge that the inclusion of this figure requires a more extensive approach from our part than what we initially saw for us. We have therefore decided to remove the image from our submission, and instead designed a table (Table 1) for the symptoms and consequences of LARS.

3. In this instance it seems there may be acceptable restrictions in place that prevent the public sharing of your minimal data. However, in line with our goal of ensuring long-term data availability to all interested researchers, PLOS’ Data Policy states that authors cannot be the sole named individuals responsible for ensuring data access (<http://journals.plos.org/plosone/s/data-availability#loc-acceptable-data-sharing-methods>).

Data requests to a non-author institutional point of contact, such as a data access or ethics committee, helps guarantee long term stability and availability of data. Providing interested researchers with a durable point of contact ensures data will be accessible even if an author changes email addresses, institutions, or becomes unavailable to answer requests.

Before we proceed with your manuscript, please also provide non-author contact information (phone/email/hyperlink) for a data access committee, ethics committee, or other institutional body to which data requests may be sent. If no institutional body is available to respond to requests for your minimal data, please consider if there any institutional representatives who did not collaborate in the study, and are not listed as authors on the manuscript, who would be able to hold the data and respond to external requests for data access? If so, please provide their contact information (i.e., email address). Please also provide details on how you will ensure persistent or long-term data storage and availability.

**RESPONSE**

Our data is qualitative and reported under results as both descriptions of the findings and quotes from participants. External requests can be sent to the following email address: [forskningsetikk@hvl.no](mailto:forskningsetikk@hvl.no)

4. When completing the data availability statement of the submission form, you indicated that you will make your data available on acceptance. We strongly recommend all authors decide on a data sharing plan before acceptance, as the process can be lengthy and hold up publication timelines. Please note that, though access restrictions are acceptable now, your entire data will need to be made freely accessible if your manuscript is accepted for publication. This policy applies to all data except where public deposition would breach compliance with the protocol approved by your research ethics board. If you are unable to adhere to our open data policy, please kindly revise your statement to explain your reasoning and we will seek the editor's input on an exemption. Please be assured that, once you have provided your new statement, the assessment of your exemption will not hold up the peer review process.

**RESPONSE**

Our data is qualitative and reported under results as both descriptions of the findings and quotes from participants. A new statement is provided.

5. Please review your reference list to ensure that it is complete and correct. If you have cited papers that have been retracted, please include the rationale for doing so in the manuscript text, or remove these references and replace them with relevant current references. Any changes to the reference list should be mentioned in the rebuttal letter that accompanies your revised manuscript. If you need to cite a retracted article, indicate the article’s retracted status in the References list and also include a citation and full reference for the retraction notice.

**RESPONSE**

We believe we now have followed your requirements for the reference list.

**Additional Editor Comments:**

1. Your made interview only five patients. Why do you make small sample?

**RESPONSE**

This study was conducted in a public county hospital and during the period of data collection, five participants who met the inclusion criteria agreed to participate. This number may be explained by the number of patients operated annually in this hospital and by only enrolling patients with sphincter preserving surgery without having an ileostomy during their disease trajectory. See line 519-521 at page 25.

2. Align your manuscript with PLOS ONE guideline

**RESPONSE**

We believe we now have followed PLOS ONE requirements for manuscripts.

**Reviewers' comments:**
Reviewer's Responses to Questions

**Comments to the Author**

1. Is the manuscript technically sound, and do the data support the conclusions?
The manuscript must describe a technically sound piece of scientific research with data that supports the conclusions. Experiments must have been conducted rigorously, with appropriate controls, replication, and sample sizes. The conclusions must be drawn appropriately based on the data presented.

Reviewer #1: Yes

Reviewer #2: Yes

**RESPONSE**

We thank the reviewers for their positive opinion of our work.

2. Has the statistical analysis been performed appropriately and rigorously?

Reviewer #1: Yes

Reviewer #2: Yes

**RESPONSE**

We thank the reviewers for their positive opinion of our work.

3. Have the authors made all data underlying the findings in their manuscript fully available?
   The [PLOS Data policy](http://www.plosone.org/static/policies.action#sharing) requires authors to make all data underlying the findings described in their manuscript fully available without restriction, with rare exception (please refer to the Data Availability Statement in the manuscript PDF file). The data should be provided as part of the manuscript or its supporting information, or deposited to a public repository. For example, in addition to summary statistics, the data points behind means, medians and variance measures should be available. If there are restrictions on publicly sharing data—e.g. participant privacy or use of data from a third party—those must be specified.

Reviewer #1: Yes

Reviewer #2: Yes

**RESPONSE**

We thank the reviewers for their positive opinion of our work.

4. Is the manuscript presented in an intelligible fashion and written in standard English? PLOS ONE does not copyedit accepted manuscripts, so the language in submitted articles must be clear, correct, and unambiguous. Any typographical or grammatical errors should be corrected at revision, so please note any specific errors here.

Reviewer #1: Yes

Reviewer #2: Yes

**RESPONSE**

We thank the reviewers for their positive opinion of our work.

4. Review Comments to the Author

Reviewer #1: It is an interesting area of research undertaken by your team gathering insight into living with LARS. My only concern is why the researchers have settled with 5 participants only to give their recommendations. Was it not possible to recruit more patients to have a broader inclusion and diverse opinions?

**RESPONSE**

This study was conducted in a public county hospital and during the period of data collection, five participants who met the inclusion criteria agreed to participate. This number may be explained by the number of patients operated annually in this hospital and by only enrolling patients with sphincter preserving surgery without having an ileostomy during their disease trajectory. See line 519-521 at page 25.

5. The description of the findings is appropriate in a good sequence.
   Apart from this limitation of a small sample. this study could be helpful in stimulating more research in the same area.

**RESPONSE**

Thank you for this feedback on the findings.

Reviewer #2: Strengths and weaknesses

1. the topic is not unique but worthy of researching

**RESPONSE**

Thank you, we agree with Reviewer # 2 that this is an interesting and valuable area that is worthy of research.

2. there are of papers in google scholar and Refseek about this topic since 2020

**RESPONSE**

We have now updated the manuscript with several research articles published after 2020. See the reference list of several papers from Pape et al.

3. Ethical approval is mentioned

**RESPONSE**

We appreciate this comment.

4. the title is too long. You can shorten it to be more attractive and citable

**RESPONSE**

We find it difficult to shorten the title, because we will then miss both the study period and the design of the study. Therefore, we want to keep the title: Patients’ experiences of living with low anterior resection syndrome **three to six months** after colorectal cancer surgery: A phenomenological study. The title length is also within the word limit.

To shorten the title, we have removed the study design. The short title length is in accordance with the word limit.

5. the abstract contains plagiarism. Please revise it. Otherwise, it is informative

**RESPONSE**

We are not able to see how the introduction and conclusion contain plagiarism. However, we have revised the result section by removing the numbers of the themes. The aim and the design will inevitably contain plagiarism.

6. the aim is clear

**RESPONSE**

We appreciate this comment.

7. the KEYWORDS are fair. Please use <http://www.ncbi.nlm.nih.gov/mesh>

**RESPONSE**

Thank you.

8. lack of the abbreviations section

**RESPONSE**

We were not able to find the abbreviations section. However, LARS- low anterior resection syndrome, is the only abbreviation in the text, but it is explained.

9. the introduction provide sufficient background information for readers in the immediate field to understand the problem/hypotheses

**RESPONSE**

We appreciate this comment.

10. the text arrangement is good

**RESPONSE**

We appreciate this comment.

11. the method section is good

**RESPONSE**

We appreciate this comment.

12. the depth of the academic material is good

**RESPONSE**

We appreciate this comment.

13. the study design is good

**RESPONSE**

We appreciate this comment.

14. The suitability and accuracy of questions is good

**RESPONSE**

We appreciate this comment.

15. The research methodology is suitable

**RESPONSE**

We appreciate this comment.

16. The materials are good

**RESPONSE**

We appreciate this comment.

17. the logic is clear

**RESPONSE**

We appreciate this comment.

18. the paper is not novel

**RESPONSE**

What is novel in our paper is the period which is studied; three to six months after surgery. Most of the earlier papers have studied the period from one year and further on.

19. there are few grammatical errors in this article

**RESPONSE**

We appreciate this comment and believe the grammar is corrected.

20. the related concepts are introduced

**RESPONSE**

We appreciate this comment.

21. the readability is sufficient

**RESPONSE**

We appreciate this comment.

22. the results are good

**RESPONSE**

We appreciate this comment.

23. all figures/tables are clear enough to summarize the results for presentation to the readers

**RESPONSE**

We appreciate this comment.

24. all figures/tables are well referred to in the text

**RESPONSE**

We appreciate this comment.

25. the theoretical analysis in this article is sufficient

**RESPONSE**

We appreciate this comment.

**26. the discussion of results from multiple angles is sufficient**

**RESPONSE**

We appreciate this comment.

27. the conclusion is tenable

**RESPONSE**

We appreciate this comment.

28. the reference section contains too many old references

**RESPONSE**

The reference list is now updated with several references after 2020. Several papers from the research group of Pape are included.

29. please use (google scholar and Refseek) search engines then set it since 2020

**RESPONSE**

We appreciate this comment and have used Refseek.

30. the references are in order within the text

**RESPONSE**

We appreciate this comment.

31. Bias is present

**RESPONSE**

We don’t understand this comment, as this is a phenomenological study. Bias is used as a concept in quantitative research. We have outlined the rigour in our qualitative study.

32. There is no conflict of interest with the author about this topic

**RESPONSE**

We appreciate this comment.

33. Fund is mentioned

**RESPONSE**

We appreciate this comment.

34. Conflict of interest is mentioned

**RESPONSE**

We appreciate this comment.

35. Acknowledgement is mentioned

**RESPONSE**

We appreciate this comment.

36. You can use my suggestions

**RESPONSE**

We appreciate your suggestions and have responded to them.

My final decision is acceptable after minor revision

**RESPONSE**

We are thankful to the Academic Editor and the reviewers for their valuable contributions and hope that our responses are to your satisfaction.

Kind regards

**Marit Hegg Reime**

Professor

Department of Health and Caring sciences

Western Norway University of Applied Sciences
