# Supplementary material for: Patients’ experiences of living with low anterior resection syndrome three to six months after colorectal cancer surgery: A phenomenological study
Source: PLoS One. 2024 Jul 10;19(7):e0305212. doi: 10.1371/journal.pone.0305212 (PMC11236105; doi:10.1371/journal.pone.0305212)
Supplement: Attachment — Submitted filename: Response to reviewers 23.05.24.docx [file pone.0305212.s004.docx]

Bergen, Norway

May 23, 2024

PONE-D-23-42953

Patients’ experiences of living with low anterior resection syndrome three to six months after colorectal cancer surgery: A phenomenological study

PLOS ONE

Atalel Fentahun Awedew, MD,MPH, Ass.Prof Surgery
Academic Editor
PLOS ONE

Dear Academic Editor

After some mail correspondence with the journal about what to edit in the revised version, it came to our understanding that it was just the heading in the abstract which required editing, in accordance with your answer in the mail from 22.05.

We have now revised the heading to Objective in the abstract, according to the following feedback:

**I extend my heartfelt appreciation to the esteemed authors for addressing all insightful comments and constructive feedback. The manuscript not only sheds light on sensitive issues in Colorectal surgery but also introduces a fresh perspective to the field. I have duly noted the minor formatting suggestions provided, including the incorporation of the study design in the methodology section and changing "aim" to "Objective." Following the suggested format of Background, Objective, Method, Results, Conclusion, and Keywords will enhance the clarity and structure of the manuscript. Please submit the clear manuscript with recommended format of abstract within 48hours.**

We are thankful to the Academic Editor and the reviewers for their valuable contributions and hope that our responses are to your satisfaction.

Kind regards

**Marit Hegg Reime**

Professor

Department of Health and Caring sciences

Western Norway University of Applied Sciences
